# Bcl2 Deficiency Activates FoxO through Akt Inactivation and Accelerates Osteoblast Differentiation

**DOI:** 10.1371/journal.pone.0086629

**Published:** 2014-01-20

**Authors:** Takeshi Moriishi, Yosuke Kawai, Hisato Komori, Satoshi Rokutanda, Yutaka Eguchi, Yoshihide Tsujimoto, Izumi Asahina, Toshihisa Komori

**Affiliations:** 1 Department of Cell Biology, Nagasaki University Graduate School of Biomedical Sciences, Nagasaki, Japan; 2 Department of Regenerative Oral Surgery, Nagasaki University Graduate School of Biomedical Sciences, Nagasaki, Japan; 3 Department of Oral and Maxillofacial Surgery, Nagasaki University Graduate School of Biomedical Sciences, Nagasaki, Japan; 4 Department of Molecular Genetics, Osaka University Medical School, Osaka, Japan; Columbia University, United States of America

## Abstract

Osteoblast apoptosis plays an important role in bone development and maintenance, and is in part responsible for osteoporosis in sex steroid deficiency, glucocorticoid excess, and aging. Although Bcl2 subfamily proteins, including Bcl2 and Bcl-XL, inhibit apoptosis, the physiological significance of Bcl2 in osteoblast differentiation has not been fully elucidated. To investigate this, we examined Bcl2-deficient (Bcl2^−/−^) mice. In Bcl2^−/−^ mice, bromodeoxyuridine (BrdU)-positive osteoblasts were reduced in number, while terminal deoxynucleotidyl transferase-mediated dUTP nick end-labeling (TUNEL)-positive osteoblasts were increased. Unexpectedly, osteoblast differentiation was accelerated in Bcl2^−/−^ mice as shown by the early appearance of *osteocalcin*-positive osteoblasts. Osteoblast differentiation was also accelerated in vitro when primary osteoblasts were seeded at a high concentration to minimize the reduction of the cell density by apoptosis during culture. FoxO transcription factors, whose activities are negatively regulated through the phosphorylation by Akt, play important roles in multiple cell events, including proliferation, death, differentiation, longevity, and stress response. Expressions of *FasL*, *Gadd45a*, and *Bim*, which are regulated by FoxOs, were upregulated; the expression and activity of FoxOs were enhanced; and the phosphorylation of Akt and that of FoxO1 and FoxO3a by Akt were reduced in Bcl2^−/−^ calvariae. Further, the levels of p53 mRNA and protein were increased, and the expression of p53-target genes, Pten and Igfbp3 whose proteins inhibit Akt activation, was upregulated in Bcl2^−/−^ calvariae. However, *Pten* but not *Igfbp3* was upregulated in Bcl2^−/−^ primary osteoblasts, and p53 induced *Pten* but not *Igfbp3* in vitro. Silencing of either *FoxO1* or *FoxO3a* inhibited and constitutively-active FoxO3a enhanced osteoblast differentiation. These findings suggest that Bcl2 deficiency induces and activates FoxOs through Akt inactivation, at least in part, by upregulating *Pten* expression through p53 in osteoblasts, and that the enhanced expression and activities of FoxOs may be one of the causes of accelerated osteoblast differentiation in Bcl2^−/−^ mice.

## Introduction

Osteoblast apoptosis plays an important role in bone development and maintenance. All major regulators of bone metabolism, including estrogen, androgen, parathyroid hormone (PTH), locally produced factors like interleukin 6 (IL-6)-type cytokine, bone morphogenetic proteins (BMPs), insulin-like growth factor-1 (IGF-1), Wnts, PTH-related peptide (PTHrP), mechanical forces, and oxidative stress, modulate osteoblast and osteocyte apoptosis [1]. It is estimated that 60–80% of osteoblasts that originally assembled at the resorption pit die by apoptosis. Further, bone loss caused by sex steroid deficiency, glucocorticoid excess, or aging is due in part to osteoblast apoptosis, and PTH, bisphosphonate, and calcitonin exert anabolic action on bone by inhibiting osteoblast and osteocyte apoptosis, [2], [3], [4], [5], [6], [7], [8], [9]. Bcl2 subfamily proteins, including Bcl2 and Bcl-XL, inhibit apoptosis through prevention of the release of caspase activators from mitochondria by inhibiting Bax subfamily proteins [10]. Thus, the bone loss caused by sex steroid deficiency, glucocorticoid excess, or aging might be inhibited by Bcl2; however, the physiological significance of Bcl2 in osteoblast differentiation and bone development and maintenance has not been fully investigated.

Activation of phosphatidylinositol 3-kinase (PI3K) by a number of growth factors, including insulin and insulin-like growth factors (IGF), results in the production of phosphatidylinositol-(3,4,5)-triphosphate (PIP3), and this in turn causes localization of the kinase Akt to the plasma membrane. At the plasma membrane, Akt can be phosphorylated by Pdk1 and mTORC2 (consisting of the kinase mTOR, Rictor, Sin1, and mLST8 complex), leading to its full activation. Activated Akt phosphorylates a subset of targets, including the FoxO family of transcription factors, which include FoxO1, FoxO3a, and FoxO4. Phosphorylated FoxO factors interact with the adaptor 14-3-3, which promotes relocalization to the cytoplasm. Oxidative stress opposes nuclear export by alternative phosphorylation of FoxO factors. Phosphorylation mediated by JNK and Mst1, which are activated by oxidative stress, promotes translocation to the nucleus [11], [12], [13]. p53, which senses various intrinsic and extrinsic stress signals, induces the negative regulators, including Igfbp3 and Pten, in the PI3K-Akt pathway to shut down cell growth and division to avoid the introduction of infidelity into the process of cell growth and division [14], [15]. Igfbp3 binds to free IGF-1 and prevents it from binding to the IGF-1 receptors, and Pten reverses the effects of PI3K by dephosphorylating PIP3 [16].

Recently, FoxO-dependent oxidative defense was shown to be important for bone formation and bone mass homeostasis [17], [18]. FoxOs inhibit osteoblast apoptosis through the suppression of oxidative stress [17]. Further, FoxO1 regulates osteoblast proliferation through the interaction with ATF4, a transcription factor regulating amino acid import, as well as through the suppression of p19ARF and p16 and downstream activation of their target protein p53 [18]. Further, FoxO1 has been shown to regulate osteoblast differentiation [19], [20].

The previous reports showed that osteoblast apoptosis was unchanged or increased, and osteoblast differentiation was unchanged or inhibited in Bcl2-deficient (Bcl2^−/−^) primary osteoblasts compared with wild-type primary osteoblasts in vitro [21], [22]. However, we found that osteoblast differentiation is inhibited in osteoblast-specific Bcl2 transgenic mice [23]. Further, we found that differentiation of the primary osteoblasts from Bcl2 transgenic mice is also inhibited in vitro, but that it is affected by apoptosis, because osteoblast apoptosis reduces cell density and leads to the deceleration of osteoblast differentiation [23]. Thus, we examined osteoblast proliferation, apoptosis, and differentiation in the bone tissues of Bcl2^−/−^ mice to evaluate the physiological roles of Bcl2 in osteoblasts. Contrary to the previous reports, osteoblast differentiation was accelerated in Bcl2^−/−^ mice. The differentiation of Bcl2^−/−^ primary osteoblasts was also accelerated in vitro, when the cells were seeded at a high concentration to minimize the reduction of the cell density by apoptosis during culture. Thus, we further pursued the mechanism of enhanced osteoblast differentiation in vivo using bone tissues. Here, we show that the deletion of Bcl2 accelerated osteoblast differentiation, at least in part, through the Akt-FoxO pathway.

## Materials and Methods

### Ethics Statement

Prior to the study, all experiments were reviewed and approved by the Animal Care and Use Committee of Nagasaki University Graduate School of Biomedical Sciences. (Permit Number: 0906170767-4).

### Animal Study

Bcl2^−/−^ mice were generated as previously described [24]. Briefly, ES cells derived from 129/Ola were injected into the blastocysts recovered from the mating of B6C3F1 (C57BL/6 x C3H F1) with C57BL/6, and the chimeric mice were mated with ICR. Wild-type, heterozygous, and homozygous mice were obtained by brother-sister mating of heterozygous mice.

### Histological Analysis

The bone histomorphometric analysis was performed by measuring the area and perimeter of trabecular bone of femurs at 2 weeks of age with Image J using the H-E stained paraffin-embedded sections. For histological analyses of the long bones, mice were sacrificed and fixed in 4% paraformaldehyde/0.01 M phosphate-buffered saline, and the long bones were decalcified in 10% EDTA (pH7.4) and embedded in paraffin. Sections (3–7 µm thick) were stained with hematoxylin and eosin (H-E) or stained for TUNEL using the ApopTag® system (Intergen, Burlington, MA), or subjected to in situ hybridization using Col1a1, osteopontin, and osteocalcin probes [25]. For the BrdU incorporation study, mice of 2 weeks of age were injected intraperitoneally with 100 µg BrdU/gram body weight and sacrificed 1 hour later. Sections were stained with the BrdU staining kit (Zymed, San Francisco, CA). In the counting of TUNEL-positive or BrdU-positive osteoblastic cells, only the cells in the distal primary spongiosa of femurs, which were recognized as osteoblastic cells from the morphology and attachment to the trabecular bone, were counted.

### Real-time RT-PCR and Western Blot Analyses

Total RNA was extracted using ISOGEN (Wako, Osaka, Japan), and real-time RT-PCR was performed as previously described [26]. Primer sequences are shown in Table S1. We normalized the values to that of *Gapdh*. Western blot analysis was performed using the following antibodies: anti-Akt, anti-phosphorylated Akt, anti-FoxO1, anti-FoxO3a, anti-phosphorylated FoxO1 (Thr24)/FoxO3a (Thr32), anti-JNK, anti-phosphorylated JNK, anti-Mst1, and anti-phosphorylated Mst1 antibodies (Cell Signaling, Danvers, MA); anti-phosphorylated FoxO3a (S207) antibody (Invitrogen, Tokyo, Japan); and anti-actin antibody (Santa Cruz Biotechnology, Santa Cruz, CA).

### In situ Hybridization

For in situ hybridization, we prepared digoxigenin-11-UTP-labeled single-stranded RNA probes using a DIG RNA labeling kit (Roche Biochemica) according to the manufacturer's instructions. We used a 0.32 kb fragment of *Col1a1* cDNA [27], a 1.2 kb fragment of mouse *osteopontin* cDNA [28], and a 0.47 kb fragment of mouse osteocalcin cDNA [29] to generate antisense and sense probes. We carried out hybridization as previously described [28] and counterstained the sections with methyl green.

### Cell Culture Experiments

Primary osteoblasts were isolated from newborn calvaria by sequential digestion with 0.1% collagenase A and 0.2% dispase. Osteoblastic cells from the third to fifth fraction were pooled, plated on 48-well plates at a density of 2.5×10^4^/well and 24-well plates at a density of 5×10^4^/well, and used for MTT, osteoblast differentiation, and TUNEL assays. To examine osteoblast differentiation, staining for alkaline phosphatase (ALP) activity and mineralization was performed as previously described [30]. Quantification of mineralization was performed using VHX-1000 (KEYENCE) and Image J. TUNEL-positive cells were detected using the ApopTag® system (Intergen, Burlington, MA). FoxO3a-AAA triple mutant (FoxO3a-TM) adenovirus was a gift from K. Walsh (Boston University Medical School) [31]. In FoxO3a-TM, the three phosphorylation sites, Thr-32, Ser-253, and Ser-315, were replaced by alanine residues. MC3T3-E1 cells were infected with the retrovirus vector (pSIREN-RetroQ, Takara Bio, Inc. Otsu, Japan) expressing each shRNA for GFP, *FoxO1*, and *FoxO3a*, and cultured for 3 days in the presence of puromycin. BMP2 (100 ng/ml) was added to the medium at confluence. A p53^−/−^ osteoblast cell line, which was established from p53^−/−^ calvarial cells, was infected with human p53-expressing retrovirus or empty retrovirus. Retrovirus was constructed by inserting full length human p53 cDNA into pDON-5 (Takara Bio, Inc.).

### Reporter Assay

Primary osteoblasts from wild-type and Bcl2^−/−^ mice were transfected with the Gadd45a promoter construct [32] and pRL-CMV by FuGENE 6 (Roche Diagnostics, Tokyo, Japan). Luciferase activity was normalized to Renilla luciferase activity using pRL-CMV (Promega, Madison, WI).

### Statistical Analysis

Statistical analyses were performed using Student's t-test. Ekuseru-Toukei 2010 (Social Survey Research Information Co., Ltd., Tokyo, Japan). Data are presented as the mean ± S.D. A P-value of less than 0.05 was considered significant.

## Results

### Increase in Bone Mass and Osteoblast Apoptosis in Bcl2^−/−^ Mice

**Figure 1:**
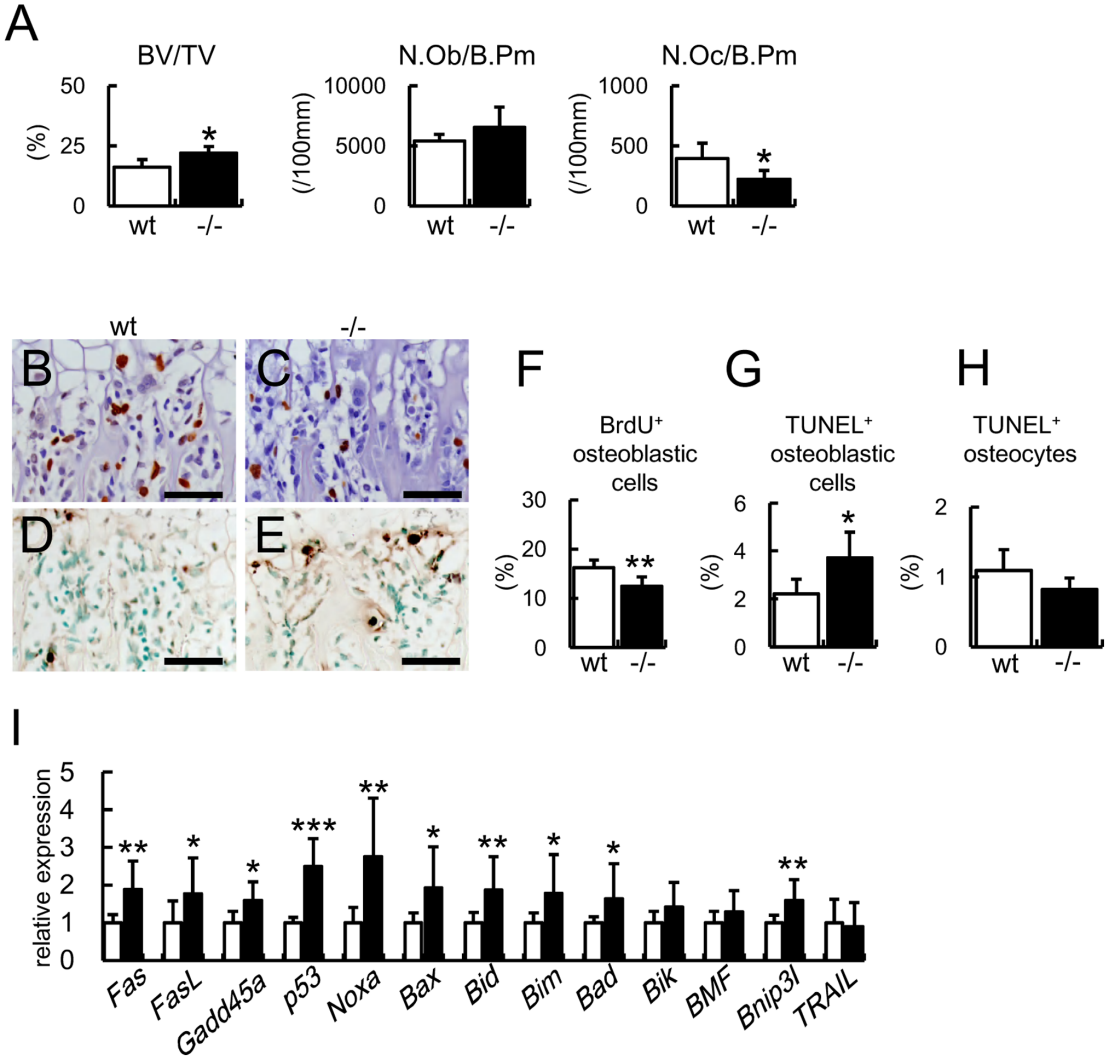
Bone morphometric analysis, BrdU and TUNEL staining, and real-time RT-PCR analysis of apoptosis-related genes in Bcl2^−/−^ mice. (A) Bone histomorphometric analysis. The trabecular bone volume (bone volume/tissue volume, BV/TV), number of osteoblasts (N.Ob/B.Pm), and number of osteoclasts (N.Oc/B.Pm) were compared in femurs between 6 wild-type and 4 Bcl2^−/−^ mice at 2 weeks of age. B.Pm, bone perimeter. (B–H) BrdU labeling (B, C) and TUNEL staining (D, E) of sections of femurs from wild-type mice (B, D) and Bcl2^−/−^ mice (C, E). Bars  = 50 µm. BrdU-positive osteoblastic cells (F), TUNEL-positive osteoblastic cells (G), and TUNEL-positive osteocytes (H) were counted and shown as a percentage of the number of osteoblastic cells or osteocytes. wild-type mice, n = 7; Bcl2^−/−^ mice, n = 5 in F. wild-type mice, n = 8; Bcl2^−/−^ mice, n = 5 in G and H. (I) Real-time RT-PCR analysis of apoptosis-related genes. RNA was directly extracted from newborn calvariae of wild-type and Bcl2^−/−^ mice. wild-type mice, n = 6; Bcl2^−/−^ mice, n = 15. *vs. wild-type mice. *P<0.05, **P<0.01.

As Bcl2^−/−^ mice died at approximately 2–3 weeks of age, bone histomorphometric analysis was performed on the trabecular bone of femurs at 2 weeks of age (Fig. 1A). The bone volume was increased in Bcl2^−/−^ mice and the density of osteoblasts in Bcl2^−/−^ mice was similar to that in wild-type mice. In contrast, the density of osteoclasts was reduced in Bcl2^−/−^ mice. The percentage of BrdU-positive osteoblastic cells in Bcl2^−/−^ mice was less than that in wild-type mice (Fig. 1B, C, F), while the percentage of TUNEL-positive osteoblastic cells was increased in Bcl2^−/−^ mice compared with wild-type mice (Fig. 1D, E, G). The percentage of TUNEL-positive osteocytes in Bcl2^−/−^ mice was similar to that in wild-type mice (Fig. 1H). The expression of apoptosis-related genes, including *Fas*, *Fas*L, *p53*, *Noxa*, *Bax*, *Bid*, *Bim*, *Bad*, *Bnip3l*, was increased in calvaria of Bcl2^−/−^ mice compared with wild-type mice (Fig. 1I).

### Osteoblast Differentiation was Accelerated in Bcl2^−/−^ Mice

**Figure 2:**
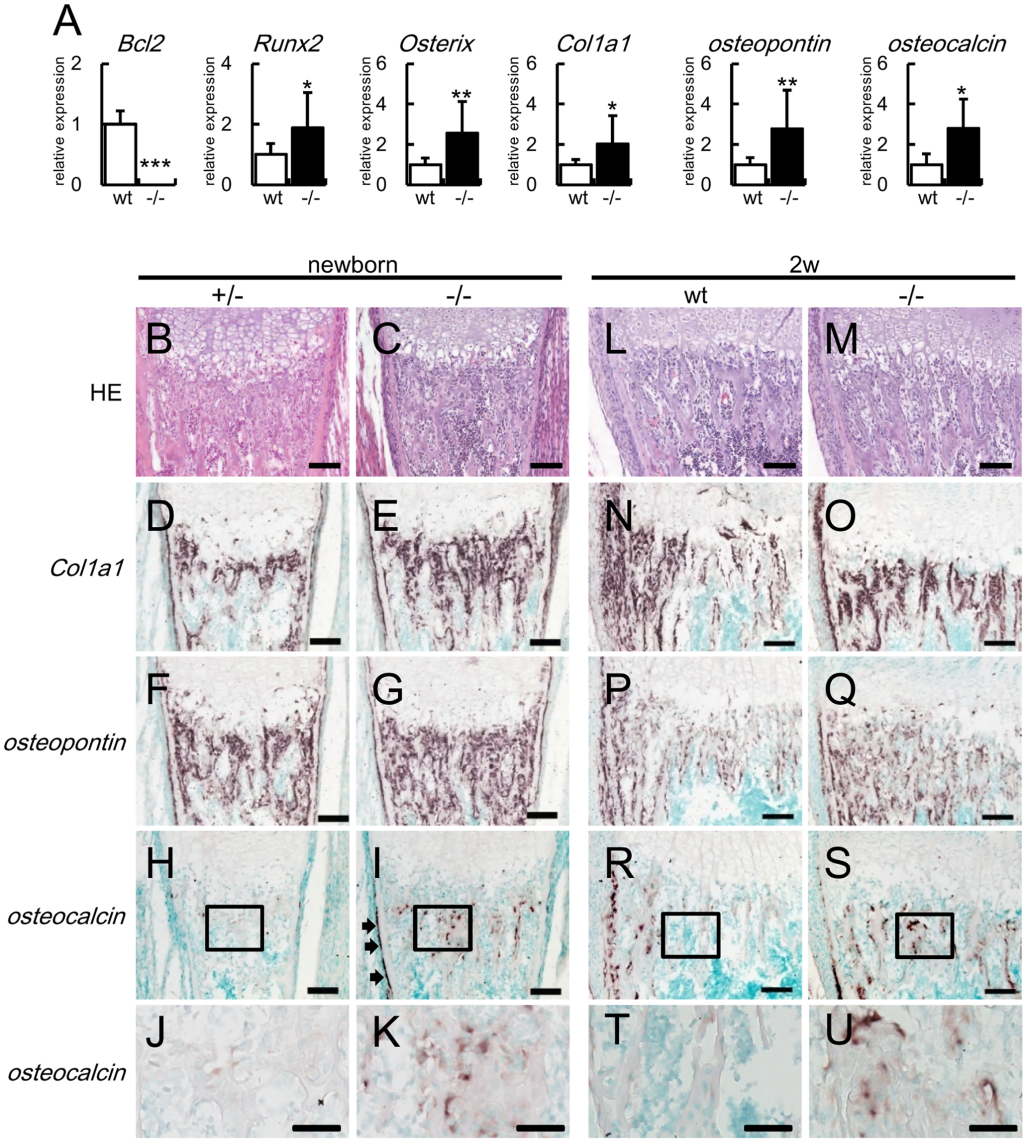
Expression of bone matrix protein genes in Bcl2^−/−^ mice. (A) Real-time RT-PCR analysis of *Bcl2*, *Runx2*, *Osterix*, *Col1a1*, *osteopontin*, and *osteocalcin*. RNA was directly extracted from newborn calvariae of wild-type (wt) and Bcl2^−/−^ mice. The values of wild-type mice were defined as 1, and relative levels are shown. wild-type mice, n = 6; Bcl2^−/−^ mice, n = 15. *vs. wild-type mice. *P<0.05, **P<0.01, ***P<0.001. (B–U) In situ hybridization analysis of *Col1a1*, *osteopontin*, and *osteocalcin*. The sections of femurs from Bcl2^+/−^ mice (B, D, F, H, J), Bcl2^−/−^ mice (C, E, G, I, K, M, O, Q, S, U), and wild-type mice (L, N, P, R, T) at birth (B–K) and at 2 weeks of age (L–U) were stained with H–E (B, C, L, M) or hybridized with *Col1a1* (D, E, N, O), *osteopontin* (F, G, P, Q), and *osteocalcin* (H–K, R–U) probes. Boxed regions in H, I, R, and S are magnified in J, K, T, and U, respectively. Arrows in I indicate the appearance of *osteocalcin*-expressing cells in the bone collar. Similar results were obtained in two newborn mice and three 2-week-old mice in each genotype and representative data are shown. In situ hybridization using the sense probes showed no significant signals (data not shown). Bars: 100 µm (B–I, L–S); 50 µm (J, K, T, U).

We examined the expression of osteoblast differentiation marker genes, including *Runx2*, *Osterix*, *Col1a1*, *osteopontin*, and *osteocalcin*, in calvariae of Bcl2^−/−^ mice by real-time RT-PCR analysis. *Runx2* and *Osterix* are upregulated in preosteoblasts, *Col1a1* and *osteopontin* are upregulated in immature osteoblasts, and *osteocalcin* is upregulated in mature osteoblasts [33], [34]. The expressions of all of these markers were increased in Bcl2^−/−^ mice compared with wild-type mice (Fig. 2A). Further, we examined osteoblast differentiation by in situ hybridization at birth and 2 weeks of age. *Col1a1*-expressing cells and *osteopontin*-expressing cells were increased at birth and 2 weeks of age in Bcl2^−/−^ mice compared with wild-type mice, reflecting the increased bone volume and similar osteoblast density compared with those in wild-type mice (Fig. 1A, 2D–G, N–Q). In wild-type mice, there were few *osteocalcin*-expressing cells and its expression level was low at birth, but both the number and expression level were increased in the bone collar and the trabecular bone near the bone collar but not in the other trabecular bone at 2 weeks of age (Fig. 2H, J, R, T). In Bcl2^−/−^ mice, however, *osteocalcin*-expressing cells were apparently present in both the bone collar and trabecular bone at birth and they were observed in the entire trabecular bone at 2 weeks of age (Fig. 2I, K, S, U). These findings indicate that osteoblast differentiation was accelerated in Bcl2^−/−^ mice.

### Proliferation, Differentiation, and Apoptosis of Bcl2^−/−^ Osteoblasts in vitro

**Figure 3:**
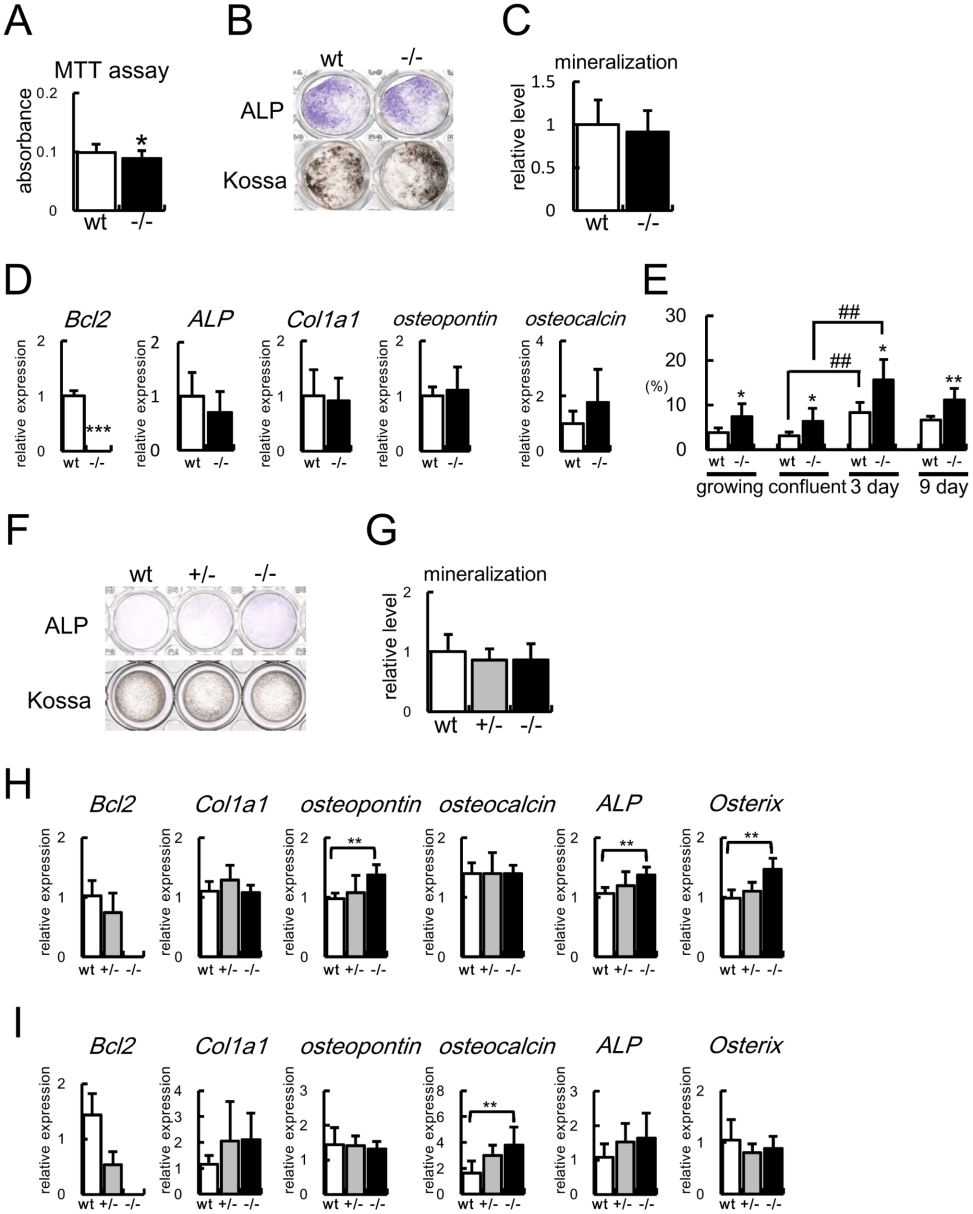
Analyses of proliferation, differentiation, and apoptosis of Bcl2−/− primary osteoblasts. (A) MTT assay. Primary osteoblasts from calvariae of 4 wild-type and 3 Bcl2^−/−^ mice were cultured for 3 days, the cells were replated, and MTT assay was performed 24 hrs later. n = 16. Similar results were obtained in two independent experiments and representative data are shown. (B–D) Differentiation of primary osteoblasts from Bcl2^−/−^ mice. Primary osteoblasts were prepared from newborn calvariae of wild-type and Bcl2^−/−^ mice, and ALP and von Kossa staining (B), quantification of mineralization (C), and real-time RT-PCR analysis (D) were performed. Primary osteoblasts were seeded at a concentration of 2.5×10^4^/cm^2^ (day 0). 50 µg/ml ascorbic acid and 10 mM β-glycerophosphate were added at day 3, ALP activity and the osteoblast marker gene expression were examined at day 6, and mineralization was examined at day 17. The value of primary osteoblasts from wild-type mice was set as 1 and the relative level is shown. n = 3. Similar results were obtained in three independent experiments and representative data are shown. (E) Frequencies of TUNEL-positive cells during culture. Primary osteoblasts from calvariae of 6 wild-type and 10 Bcl2^−/−^ mice were stained for TUNEL before confluence, at confluence, and at 3 and 9 days after confluence. n = 4−5. Similar results were obtained in two independent experiments and representative data are shown. (F–I) Differentiation of primary osteoblasts from calvariae of Bcl2^−/−^ mice. Primary osteoblasts were seeded at a concentration of 2×10^5^/cm^2^ (day 0), 50 µg/ml ascorbic acid and 10mM β-glycerophosphate were added at day 1, ALP activity was examined at day 2 (F), mineralization was examined at day 9 (G), and the osteoblast marker gene expression was examined at day 2 (H) and 9 (I) by real-time RT-PCR. n = 7 in G; n = 5 in H; n = 10−12 in I. Similar results were obtained in three independent experiments and representative data are shown. *vs. wild-type primary osteoblasts. *P<0.05; **, ^##^P<0.01; ***p<0.001.

MTT assays showed that proliferation of Bcl2^−/−^ osteoblasts was reduced compared with that of wild-type osteoblasts (Fig. 3A). Primary osteoblasts isolated from Bcl2^−/−^ mice were seeded at a concentration of 2.5×10^4^/cm^2^, ALP activity and the osteoblast marker gene expression were examined after 6 days, and mineralization was examined after 17 days (Fig. 3B–D). The ALP activity, mineralization, and the expression of *ALP*, *Col1a1*, *osteopontin*, and *osteocalcin* were similar to those from wild-type mice (Fig. 3B–D). However, apoptosis of the proliferating osteoblasts should affect the results of the MTT assay. Further, apoptosis during culture should affect osteoblast differentiation, because osteoblast differentiation in vitro is largely dependent on the cell density [23]. Thus, we examined apoptosis during osteoblast proliferation and differentiation in vitro. Osteoblast apoptosis was significantly increased not only during proliferation but also during differentiation in Bcl2^−/−^ osteoblasts (Fig. 3E).

To minimize the reduction of cell density by apoptosis, primary osteoblasts isolated from wild-type, Bcl2^+/−^, and Bcl2^−/−^ mice were seeded at a higher concentration (2×10^5^/cm^2^) and ALP activity and the osteoblast marker gene expression were examined after 2 days (Fig. 3F, H). ALP activity and the expression of *osteopontin*, *ALP*, and *Osterix* were increased in Bcl2^−/−^ primary osteoblasts compared with those in wild-type primary osteoblasts. After 8 days, the mineralization was similar between wild-type and Bcl2^−/−^ primary osteoblasts, but *osteocalcin* mRNA was increased in Bcl2^−/−^ primary osteoblasts (Fig. 3F, G, I). Although ALP activity was slightly increased in Bcl2^+/−^ primary osteoblasts compared with wild-type primary osteoblasts, the mineralization and the osteoblast marker gene expression were similar between Bcl2^+/−^and wild-type primary osteoblasts (Fig. 3F–I).

### Upregulation and Activation of FoxOs in Bcl2^−/−^ Calvariae

**Figure 4:**
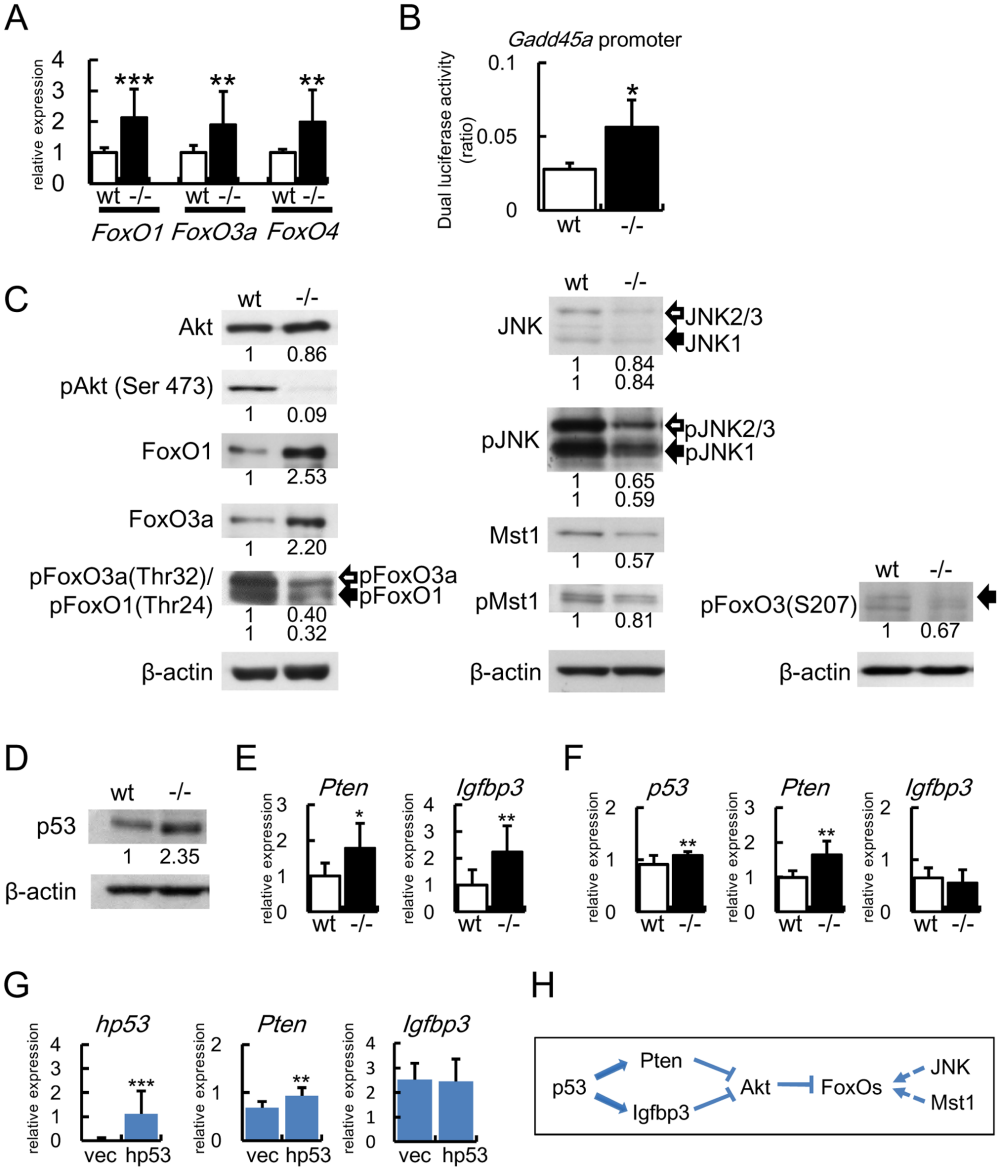
Expression and activation of FoxOs in Bcl2−/− calvariae. (A) Real-time RT-PCR analysis of the expression of FoxOs. RNA was directly extracted from newborn calvariae of wild-type and Bcl2^−/−^ mice. wild-type mice, n = 6; Bcl2^−/−^ mice, n = 15. *vs. wild-type mice, **p<0.01, ***p<0.001. (B) Reporter assay of Gadd45a promoter using wild-type and Bcl2^−/−^ primary osteoblasts. Similar results were obtained in two independent experiments and representative data are shown. (C, D) Western blot analysis. Protein was extracted from newborn calvariae of wild-type and Bcl2^−/−^ mice. The intensities of the bands were normalized against each β-actin, the normalized values in wild-type mice were set as 1, and relative levels are shown. Similar results were obtained in three independent experiments and representative data are shown. (E) Real-time RT-PCR analysis. RNA was directly extracted from calvariae of wild-type and Bcl2^−/−^ newborn mice. wild-type mice, n = 6; Bcl2^−/−^ mice, n = 15. *vs. wild-type mice. *P<0.05, **P<0.01. (F) *p53*, *Pten*, and *Igfbp3* expression in primary osteoblasts. The cDNA in Fig. 3I was used for real-time PCR analysis. n = 10−12. *vs. wild-type primary osteoblasts. **P<0.01. (G) Induction of *Pten* by p53. p53^−/−^ osteoblasts were infected with p53-expressing retrovirus or empty retrovirus. Next day, the cells were plated at the concentration of 1.5×10^5^/well in 48 well plates (day 0). 50 µg/ml ascorbic acid and 10mM β-glycerophosphate were added at day 1, and mRNA was extracted at day 4. The expression of *p53*, *Pten*, and *Igfbp3* was examined by real-time RT-PCR. Similar results were obtained in two independent experiments and representative data are shown. n = 12−13. *vs. empty retrovirus. **P<0.01, ***p<0.001. (H) Schematic presentation of the signaling pathway for FoxO activation. p53 induces Pten mRNA and Igfbp3 mRNA. Pten and Igfbp3 inhibit Akt activation. Akt inhibits the activation of FoxOs. Activation of JNK and Mst1 activate FoxOs. p53 failed to induce Igfbp3 in vitro (G). Dotted arrows indicate that the activation did not occur in Bcl2^−/−^ mice (C).

As Bcl2-deficiency enhanced osteoblast differentiation in vivo, we examined the mechanism of the accelerated osteoblast differentiation in vivo by directly analyzing the newborn calvariae. The expressions of *FasL*, *Gadd45a*, and *Bim*, which are regulated by FoxOs, were upregulated in Bcl2^−/−^ calvariae (Fig. 1I). As FoxO1 enhances osteoblast differentiation [19], [20], FoxOs might be involved in enhanced osteoblast differentiation in Bcl2^−/−^ mice. Thus, we first examined the expression and activity of FoxOs. The expressions of *FoxO1*, *FoxO3a*, and *FoxO4* mRNAs were increased in Bcl2^−/−^ calvariae compared with wild-type calvariae, and the promoter activity of Gadd45a was enhanced in Bcl2^−/−^ primary osteoblasts compared with wild-type primary osteoblasts (Fig. 4A, B). FoxO proteins are inactivated through the phosphorylation by Akt. Akt itself is activated by phosphorylation (Fig. 4H) [11], [12], [13]. Thus, we examined the activation state of Akt and FoxOs by examining their phosphorylation. The phosphorylation of Akt was markedly reduced in Bcl2^−/−^ calvariae compared with wild-type calvariae, although similar levels of Akt protein were detected (Fig. 4C). Protein levels of FoxO1 and FoxO3a were increased, whereas the phosphorylation of Thr24 in FoxO1 and that of Thr32 in FoxO3, which are phosphorylated by Akt, were reduced (Fig. 4C). These findings indicate that FoxO proteins were activated by the inactivation of Akt. FoxO proteins are also activated through the phosphorylation by JNK and Mst1, and JNK and Mst1 are activated by phophorylation (Fig. 4H) [11], [12]. Protein levels of JNK and Mst1 and the levels of their phosphorylated forms were mildly reduced, and the phosphorylation of S207 in FoxO3a, which is phosphorylated by JNK and Mst1, was also mildly reduced in Bcl2^−/−^ calvariae compared with wild-type calvariae. These findings indicate that FoxO proteins were not activated by JNK and Mst1. These findings indicate that FoxO proteins were activated in Bcl2^−/−^ primary osteoblasts through the reduction in Akt phosphorylation but not through the increase in JNK and Mst1 phosphorylation (Fig. 4H).

We further examined why Akt phosphorylation was inhibited in Bcl2^−/−^ calvariae. p53 has been shown to induce the expression of Pten and Igfbp3, whose proteins inhibit Akt phosphorylation (Fig. 4H) [14], [15], [16]. As *p53* mRNA expression was increased in Bcl2^−/−^ calvariae (Fig. 1I), we confirmed that the protein level of p53 was also increased in Bcl2^−/−^ calvariae (Fig. 4D). Further, *Pten* and *Igfbp3* expression was increased in Bcl2^−/−^ calvariae (Fig. 4E).

In the culture of primary osteoblasts, the expression of *p53* and *Pten* but not *Igfbp3* was increased in Bcl2^−/−^ primary osteoblasts compared with those in wild-type primary osteoblasts (Fig. 4F). To examine whether p53 induces Pten and Igfbp3, we introduced p53 to p53^−/−^ osteoblast cell line. *Pten* mRNA but not *Igfbp3* mRNA was induced by p53 (Fig. 4G). Further, *FoxO3a* mRNA was not induced by p53 (data not shown). These findings suggest that Akt phosphorylation was reduced, at least in part, by the induction of Pten through upregulated p53 (Fig. 4C–H).

### FoxOs Enhanced Osteoblast Differentiation

**Figure 5:**
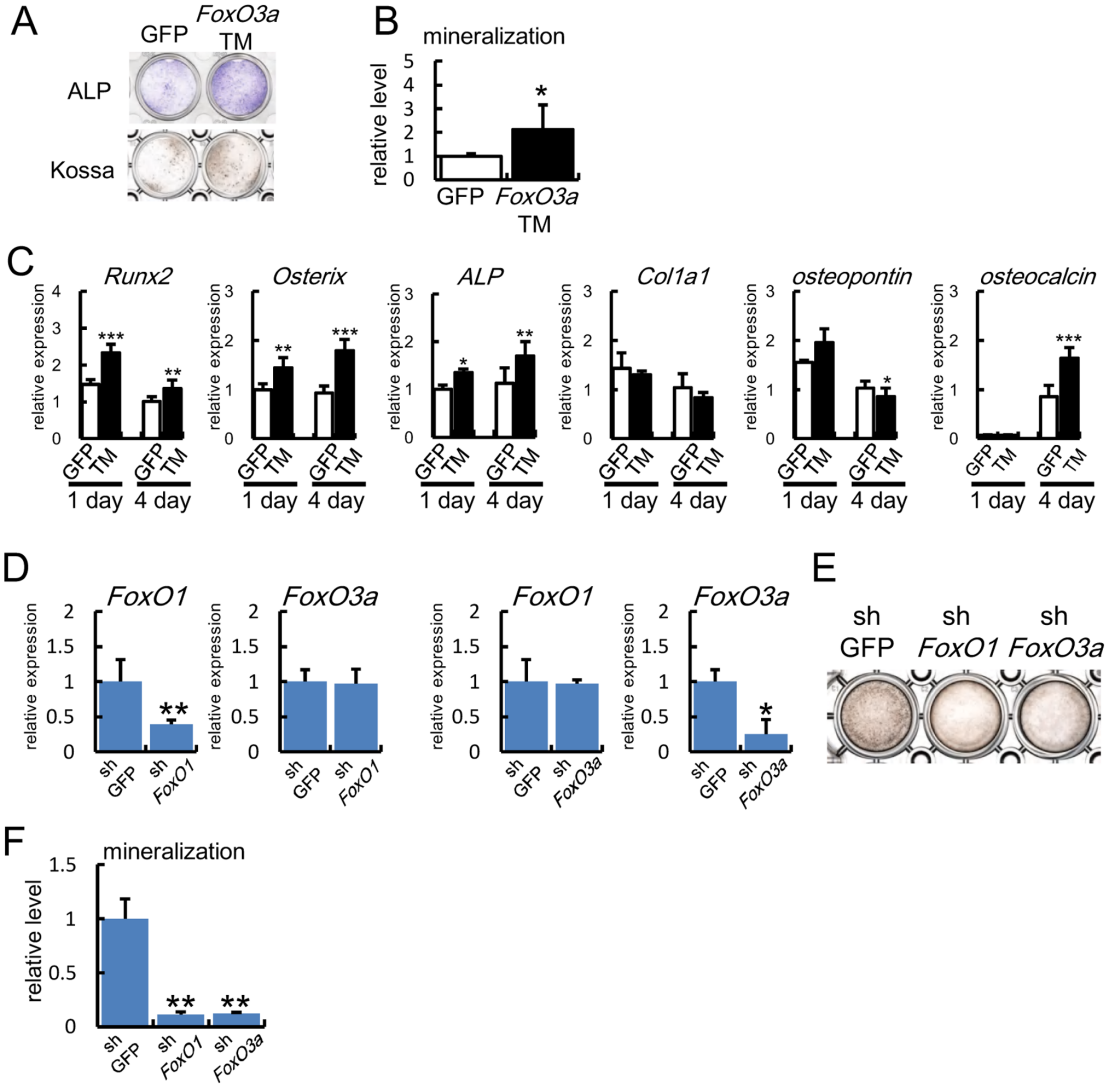
Induction of osteoblast differentiation by FoxOs. (A–C) Induction of osteoblast differentiation by FoxO3aTM. Primary osteoblasts from calvariae of wild-type mice were infected with adenovirus expressing GFP or FoxO3aTM, and ALP staining at 2 days and von Kossa staining at 6 days after infection (A), quantification of mineralization (B), and osteoblast marker gene expression (C) are shown. The value in GFP-introduced cells was set as 1 and the relative level is shown in B. Similar results were obtained in three independent experiments and representative data are shown. (D–F) Inhibition of the mineralization of MC3T3-E1 cells by sh*FoxO1* and sh*FoxO3a*. MC3T3-E1 cells were infected with retrovirus expressing GFP, sh*FoxO1*, or sh*FoxO3a*, and cultured in the presence of BMP2 (100ng/ml). The expression of *FoxO1* and *FoxO3a* was examined by real-time RT-PCR (D) and mineralization was examined by von Kossa staining (E) and its quantification (F) after culture for 2 weeks. The value in shGFP-introduced cells was set as 1 and the relative levels are shown in F. Similar results were obtained in three independent experiments and representative data are shown.

To examine whether FoxOs are able to enhance osteoblast differentiation, a constitutively active form of FoxO3a (FoxO3a-TM) was introduced into primary osteoblasts (Fig. 5A–C). FoxO3a-TM enhanced ALP activity, mineralization, and the expression of *Runx2*, *Osterix*, *ALP*, and *osteocalcin*. Further, retroviral introduction of shRNA of either *FoxO1* or *FoxO3a* into MC3T3-E1 cells reduced mineralization (Fig. 5D–F). These findings suggest that FoxOs may be involved in the enhanced osteoblast differentiation in Bcl2^−/−^ mice.

## Discussion

The proliferation was reduced and apoptosis was increased, but the differentiation was accelerated in osteoblasts and mature osteoblasts were increased in Bcl2^−/−^ mice. Therefore, our findings indicate that bone mass was increased in Bcl2^−/−^ mice not only due to the decrease in osteoclast number but also due to the acceleration of osteoblast differentiation.

The finding that osteoblast differentiation was accelerated in Bcl2^−/−^ mice was unexpected, because previous reports indicated that osteoblast differentiation was unaffected or inhibited in Bcl2^−/−^ mice based on the data of in vitro differentiation of Bcl2^−/−^ osteoblasts [21], [22]. The culture of primary osteoblasts seeded at the concentration of 2.5×10^4^/cm^2^ also showed that the differentiation of Bcl2^−/−^ osteoblasts was unaffected in vitro. However, the culture of primary osteoblasts seeded at the higher concentration (2×10^5^/cm^2^) showed that the differentiation of Bcl2^−/−^ osteoblasts was accelerated. We recently reported that overexpression of Bcl2 inhibits osteoblast differentiation in vivo and in vitro [23]. However, the inhibition of osteoblast differentiation by over-expressed Bcl2 in vitro was dependent on the cell density seeded, because overexpression of Bcl2 enhanced osteoblast differentiation by increasing cell density through the inhibition of apoptosis in vitro [23]. Therefore, the discrepancy in osteoblast differentiation in Bcl2^−/−^ mice between our data and previous reports was likely to be explained by the reduction in the cell density during culture due to the increased apoptosis in Bcl2^−/−^ osteoblasts. Indeed, we cannot completely exclude the possibility that the decreased number and dysfunction of osteoclasts in Bcl2^−/−^ mice indirectly affected the osteoblast differentiation rather than in a cell autonomous manner.

In Bcl2^−/−^ calvariae, mRNAs for *FoxO1*, *FoxO3a*, *FoxO4*, and their target genes, including *FasL*, *Gadd45a*, and *Bim*, were upregulated, and the promoter activity of Gadd45a was enhanced in Bcl2^−/−^ primary osteoblasts. Further, the phosphorylation of FoxO1 and FoxO3a by Akt was reduced due to the suppression of Akt, at least in part, through the upregulation of *Pten* and *Igfbp3*, while the phosphorylation of FoxO3a by JNK and Mst1 was not enhanced, suggesting that FoxOs were activated in Bcl2^−/−^ osteoblasts through the PI3K-Akt signaling pathway. As Pten and Igfbp3 are target genes of p53 [14], [15], the activation of FoxOs by Akt may be dependent on p53 but not Bcl2 itself. The expressions of *Pten* and *Igfbp3* were upregulated in Bcl2^−/−^ calvarial tissues, whereas the expression of *Pten* but not *Igfbp3* was upregulated in Bcl2^−/−^ primary osteoblasts. Further, introduction of p53 induced the expression of *Pten* but not *Igfbp3*. These findings indicate that upregulation of p53 is sufficient for Pten induction in vivo and in vitro, but that it is not sufficient for Igfbp3 induction in vitro. Therefore, the molecules, which cooperate with p53 for Igfbp3 induction, may be insufficient in vitro. Indeed, it is possible that other cell types including lymphocytes, in which apoptosis is accelerated [24], [43], contributed to the induction of *Igfbp3* in Bcl2^−/−^ calvarial tissues. p53 also inhibits FoxO3a activity by inducing SGK, by directly inhibiting the transcriptional activity, or by inducing FoxO3a degradation through Mdm2 [35], [36], [37]. Therefore, p53 seems to regulate FoxO activity positively or negatively depending on the cell type and cell conditions. We also showed the transcriptional upregulation of *FoxOs* in Bcl2^−/−^ calvariae. Recently, it has been shown that FoxO3a is a target gene of p53 [38], [39]. Further, FoxO1 and FoxO4 genes are regulated by FoxO3a [40]. Therefore, the increased p53 may be responsible for the upregulation of *FoxO1*, *FoxO3a*, and *FoxO4* mRNA expression in Bcl2^−/−^ calvariae. However, the introduction of p53 failed to induce *FoxO3a* mRNA in vitro (data not shown). Therefore, the mechanism of the increase of FoxOs mRNA in Bcl2^−/−^ mice still remains to be clarified.

p53 has been shown to inhibit osteoblast differentiation [41], [42]. However, it is evident in vitro but not in vivo, because the calvarial bone volume is mildly reduced in p53^−/−^ mice compared with wild-type mice [41]. Since the deletion of p53 enhances proliferation and inhibits apoptosis, p53 deletion should increase the cell density in culture, leading to the acceleration of osteoblast differentiation in vitro, because osteoblast differentiation is dependent on the cell density in vitro [23]. Similarly, the increase in osteoblast number due to increased proliferation and reduced apoptosis should also lead to an increase in bone formation in p53^−/−^ mice as previously reported [41]. Therefore, the function of p53 in osteoblast differentiation needs to be further investigated.

Although osteoblast proliferation was not examined in vivo in previously reported Bcl2^−/−^ mice [21], [22], we showed that the number of proliferating osteoblasts was reduced in Bcl2^−/−^ mice. Further, we observed a reduction in the number of Bcl2^−/−^ primary osteoblasts in the MTT assay, suggesting that Bcl2 enhances osteoblast proliferation. However, it could also have been caused by increased apoptosis during culture. Previous reports showed that Bcl2 inhibits cell proliferation by facilitating G0 arrest and delaying G0 to S phase transition in hematopoietic cells and fibroblasts [44], and various groups showed that p27 as well as p130 was elevated in Bcl2-overexpressing cells during arrest [45], [46], [47], [48], although overexpression of Bcl2 in myocytes promoted proliferation [49]. Thus, it is possible that the decrease in proliferating osteoblasts in Bcl2^−/−^ mice was mostly a reflection of enhanced osteoblast differentiation, although the activation of FoxOs should have affected both proliferation and differentiation of osteoblasts in Bcl2^−/−^ mice [50].

In summary, osteoblast differentiation was enhanced in Bcl2^−/−^ mice, at least in part, through FoxOs. FoxOs were activated through the suppression of Akt, at least in part, by upregulation of Pten through p53. Although osteoblast apoptosis is in part responsible for osteoporosis in sex steroid deficiency, glucocorticoid excess, and aging, our findings suggest that the stresses toward apoptosis may have a positive effect on osteoblast differentiation.

## Supporting Information

Table S1 Primer list.
(XLSX)
